# Up-Regulation of S100A8 and S100A9 in Pulmonary Immune Response Induced by a *Mycoplasma capricolum* subsp. *capricolum* HN-B Strain

**DOI:** 10.3390/ani14142064

**Published:** 2024-07-14

**Authors:** Zhenxing Zhang, Xiangying Chen, Yong Meng, Junming Jiang, Lili Wu, Taoyu Chen, Haoju Pan, Zizhuo Jiao, Li Du, Churiga Man, Si Chen, Fengyang Wang, Hongyan Gao, Qiaoling Chen

**Affiliations:** Hainan Key Lab of Tropical Animal Reproduction, Breeding and Epidemic Disease Research, Animal Genetic Engineering Key Lab of Haikou, School of Tropical Agriculture and Forestry, Hainan University, Haikou 570228, China; zxzhang23@163.com (Z.Z.); xychen915@163.com (X.C.); alpha.meng@outlook.com (Y.M.); jmm992847@163.com (J.J.); wll6222023@163.com (L.W.); 18889542406@163.com (T.C.); haoju36999@163.com (H.P.); jiaozizhuo1998@outlook.com (Z.J.); kych2008dl@163.com (L.D.); manchuriga@163.com (C.M.); chensi.ruth@hotmail.com (S.C.); fywang68@163.com (F.W.)

**Keywords:** *Mycoplasma capricolum* subsp. *capricolum*, RNA sequencing, immunohistochemistry, S100A8, S100A9

## Abstract

**Simple Summary:**

*Mycoplasma capricolum* subsp. *capricolum* (Mcc) is an important pathogen associated with diseases in goats, hindering the development of the livestock industry. We previously isolated the HN-B strain of Mcc from goats on Hainan Island, China. The genomic and biological characteristics of this strain were investigated. In this study, we infected mice and RAW264.7 cells with Mcc HN-B to explore its pathogenic mechanism in the host. Differentially expressed genes related to immune terms and pathways were identified using bioinformatic analyses. The expression of two inflammatory proteins, S100A8 and S100A9, was up-regulated in mouse lungs post Mcc HN-B infection. This study preliminarily elucidates the pathogenesis of Mcc in mice and paves the way for further research in goats.

**Abstract:**

*Mycoplasma capricolum* subsp. *capricolum* (Mcc), a member of the *Mycoplasma mycoides* cluster, has a negative impact on the goat-breeding industry. However, little is known about the pathogenic mechanism of Mcc. This study infected mice using a previously isolated strain, Mcc HN-B. Hematoxylin and eosin staining, RNA sequencing, bioinformatic analyses, RT-qPCR, and immunohistochemistry were performed on mouse lung tissues. The results showed that 235 differentially expressed genes (DEGs) were identified. GO and KEGG enrichment analyses suggested that the DEGs were mainly associated with immune response, defensive response to bacteria, NF-kappa B signaling pathway, natural killer cell-mediated cytotoxicity, and T cell receptor signaling pathway. RT-qPCR verified the expression of *Ccl5*, *Cd4*, *Cd28*, *Il2rb*, *Lck*, *Lat*, *Ptgs2*, *S100a8*, *S100a9*, and *Il-33*. The up-regulation of S100A8 and S100A9 at the protein level was confirmed by immunohistochemistry. Moreover, RT-qPCR assays on Mcc HN-B-infected RAW264.7 cells also showed that the expression of *S100a8* and *S100a9* was elevated. S100A8 and S100A9 not only have diagnostic value in Mcc infection but also hold great significance in clarifying the pathogenic mechanism of Mcc. This study preliminarily elucidates the mechanism of Mcc HN-B-induced lung injury and provides a theoretical basis for further research on Mcc–host interactions.

## 1. Introduction

*Mycoplasma capricolum* subsp. *capricolum* (Mcc) belongs to the *Mycoplasma mycoides* cluster. It has no cell wall and is a prokaryotic microorganism that can pass through bacterial filters. Mcc infects goats, sheep, cows, camels, and humans [1,2,3]. However, the effectiveness of antibiotic treatment is poor. Mcc has a wide geographical distribution. Related cases have been reported in Spain, Tajikistan, Portugal, Greece, China, Australia, and the United States [4,5,6,7,8,9,10,11,12]. Moreover, there have also been reports of Mcc in regions such as Chongqing, Hainan, and Fujian in China [4,12,13].

Mcc is associated with arthritis, mastitis, keratoconjunctivitis, sepsis, and respiratory diseases in small ruminants. It is also one of the main pathogens responsible for contagious agalactia [14]. In goats infected with Mcc, acute diffuse interstitial pneumonia and diffuse accumulation of monocytes and macrophages in the alveolar wall blood vessels are observed, which result in pulmonary congestion and edema [15].

The heterodimer S100A8/A9, also known as calcium-protecting protein, is passively or actively released by neutrophils or monocytes. It exerts its effector function mainly by binding to Toll-like receptor 4 (TLR4) and the receptor for advanced glycation end products (RAGE) [16,17]. Since the release of inflammation-induced S100A8/A9 occurs earlier than leukocyte recruitment [18], it can be used as a diagnostic biomarker and therapeutic target for inflammation-related diseases and, therefore, has clinical application potential [18].

To further investigate the pathogenicity of Mcc and explore the immune response during Mcc invasion, this study infected mice with Mcc HN-B. RNA sequencing (RNA-seq) analysis and histopathological examination revealed the lung injury mechanism induced by Mcc HN-B. This revealed key pathways of host lung recognition and defense against Mcc infection, which paved the way for further studies on Mcc–host interactions.

## 2. Materials and Methods

### 2.1. Experimental Strains, Cells, and Animals

Mcc HN-B was isolated and preserved in our laboratory [12]. RAW264.7 cells were purchased from Kunming Cell Bank, Chinese Academy of Sciences (Kunming, China). Specific pathogen-free female Kunming mice weighing approximately 18–22 g were purchased from Hunan SJA Laboratory Animal Co., Ltd. (Changsha, China). This experimental protocol was reviewed and approved by the Hainan University Institutional Animal Use and Care Committee under the ethical approval code HNUAUCC-2023-00209.

### 2.2. Preparation of Mcc HN-B

Mcc HN-B was cultured as described previously [12]. Briefly, a single colony grown from glycerol stock was inoculated into a mycoplasma liquid medium for extended cultivation. The rejuvenated bacterial solution was centrifuged at 4 °C and 13,400× *g* for 20 min. The pellet was resuspended and washed thrice with sterile phosphate buffer solution (PBS). A color-change unit (CCU) assay was used to measure the titer of Mcc HN-B, after which the bacterial solution was adjusted to 5 × 10^9^ CCU/mL.

### 2.3. Mcc HN-B Infection in Mice

Twelve mice were randomly and evenly divided into a challenge (HN-B) group and a negative control (NC) group. After 3 days of feeding, the mice in the HN-B group were all intraperitoneally injected with 0.2 mL of bacterial solution at a titer of 5 × 10^9^ CCU/mL. Each mouse in the NC group was injected with the same volume of PBS as the HN-B group. Upon injection, the body condition of the mice was closely observed and recorded. When impending death occurred, the mice were euthanized, and samples were collected as described below. If the challenged mice did not die on the 3rd day, samples were collected after the mice were uniformly euthanized.

### 2.4. Histopathological Examination

To explore the histopathological changes induced by Mcc in mouse lungs, 3 mice from both the HN-B group and the NC group were euthanized, and their lungs were promptly collected. The lungs were fixed in 4% paraformaldehyde for over 48 h. After dehydration and vitrification, the lungs were immersed and embedded in melted paraffin. When the embedded lungs became cold and hard, they were sliced into 4 μm-thick sections and then dewaxed using xylene. Next, the sections were stained using a hematoxylin and eosin (H&E) dyeing kit (Servicebio, Wuhan, China). Finally, after dehydration and sealing, histopathological changes in the mouse lungs were observed.

### 2.5. RNA Extraction, Quantification, and Sequencing

To investigate the pulmonary transcriptome profile of mice after Mcc infection, 3 lung tissues from both the HN-B group and the NC group were collected under RNase-free conditions. Total RNA was extracted using TRIzol (Life Technologies, Carlsbad, CA, USA). NanoDrop 2000 (Thermo Fisher Scientific, Wilmington, DE, USA) and an Agilent Bioanalyzer 2100 system (Agilent Technologies, Santa Clara, CA, USA) were used to evaluate the quality of RNA. Subsequently, mRNA was enriched by magnetic beads with oligo (dT). Then, it was fragmented and reverse-transcribed to synthesize cDNA. The cDNA was subjected to end repair and adapter ligation. Eventually, through PCR amplification, a paired-end library (read length: 150 bp) was generated for sequencing.

### 2.6. DEG Identification and Analysis

The clean data generated from the NovaSeq 6000 platform (Illumina, San Diego, CA, USA) were aligned to the Mus musculus reference genome (accession number: GRCm38_release79.genome.fa) using HISAT2 [19]. The matched reads were assembled into complete transcripts using StringTie [20]. To evaluate gene expression levels, the fragments per million kilobases (FPKM) of each transcript were calculated using StringTie. According to the standards of |Fold Change, FC| ≥ 2 and false discovery rate (FDR) < 0.05, differentially expressed genes (DEGs) between the HN-B group and the NC group (HN-B vs. NC) were identified using DESeq2 [21]. Gene ontology (GO) and Kyoto Encyclopedia of Genes and Genomes (KEGG) enrichment analyses were conducted through a hypergeometric distribution test. Gene set enrichment analysis (GSEA) was performed on the BMKCloud (https://www.biocloud.net, accessed on 11 March 2023). Protein–protein interaction (PPI) networks were predicted on STRING (https://cn.string-db.org/, accessed on 12 March 2023) and constructed using Cytoscape (v3.9.1).

### 2.7. RT-qPCR Validation of DEGs

Total RNA was extracted from the lungs of the mice. A total of 22 genes were randomly selected from the identified DEGs to perform real-time fluorescence quantitative PCR (RT-qPCR). Primers were designed using Primer-BLAST (https://www.ncbi.nlm.nih.gov/tools/primer-blast, accessed on 16 March 2023) and are listed in Table 1. All quantitative experiments were conducted with biological and technical triplicates. The relative expressions were measured by taking *β-actin* as the reference gene [22]. The RNA-seq and RT-qPCR results of the selected genes were graphed using GraphPad Prism (v8.0.2). The equation F = 2^−(∆∆CT)^ was used to calculate the ratio of target genes to reference genes as the relative expression level of the genes. The data from RT-qPCR are presented as the mean ± standard deviation (SD).

### 2.8. Immunohistochemistry

Immunohistochemistry (IHC) was performed on the paraffin-embedded lung tissues. After antigen retrieval and inactivation of endogenous peroxidases, the 4 μm thick sections were blocked using bovine serum albumin (Servicebio, Wuhan, China). Subsequently, rabbit-derived IgG primary antibodies S100A8 (Servicebio, Wuhan, China) and S100A9 (Servicebio, Wuhan, China) were added at a dilution of 1:500 and incubated at 4 °C overnight. Next, the sections were reacted with horseradish peroxidase-conjugated secondary antibodies (goat anti-rabbit IgG, 1:200 dilution, Servicebio, Wuhan, China) at room temperature for 50 min. For the development of the color of the target proteins, 3,3′-diaminobenzidine was added. After staining the nuclei with hematoxylin, the sections were dehydrated, sealed, and observed under a white-light microscope.

### 2.9. In Vitro Cell Experiments

RAW264.7 cells were cultured at 37 °C and 5% CO_2_ in Dulbecco′s modified Eagle medium (Boster Bio, Pleasanton, CA, USA) containing 10% (*v*/*v*) fetal bovine serum (Thermo Fisher Scientific, Waltham, MA, USA). When the confluence reached 90%, Mcc HN-B was used to infect RAW264.7 cells (multiplicity of infection, MOI = 1). Meanwhile, uninfected RAW264.7 cells were parallelly cultured as the blank control. Cells were harvested at 36 h post-infection (h.p.i.). Total RNA was prepared using TRIzol and reverse-transcribed into cDNA. The expression levels of *S100a8* and *S100a9* were measured with RT-qPCR as described above. The quantitative results were plotted using GraphPad Prism.

## 3. Results

### 3.1. Histopathological Observation

**Figure 1:**
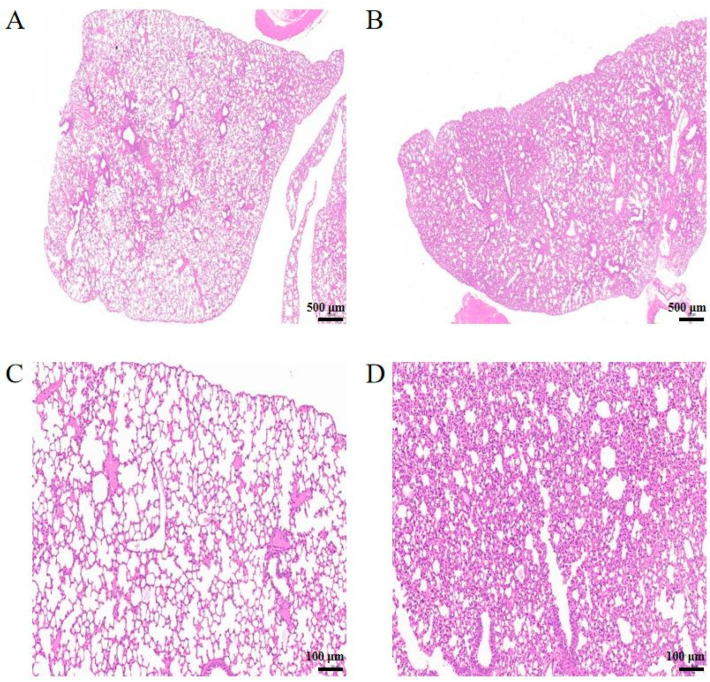
H&E staining of mouse lungs from the HN-B group and the NC group. (**A**) Lungs of mice in the NC group, scale bar = 500 µm; (**B**) lungs of mice in the HN-B group, scale bar = 500 µm; (**C**) lungs of mice in the NC group, scale bar = 100 µm; (**D**) lungs of mice in the HN-B group, scale bar = 100 μm.

H&E staining showed that the alveolar structure of the lungs in the NC group was clear and uniform without exudate. Occasionally, macrophages and lymphocytes infiltrated around the alveolar septum, blood vessels, or bronchi (Figure 1A,C). However, the alveolar septum of the lungs in the HN-B group was significantly widened, and a large amount of exudate was present in the alveoli. Additionally, a substantial number of lymphocytes and macrophages infiltrated around the alveolar septum, blood vessels, or bronchi (Figure 1B,D).

### 3.2. DEG Identification

**Figure 2:**
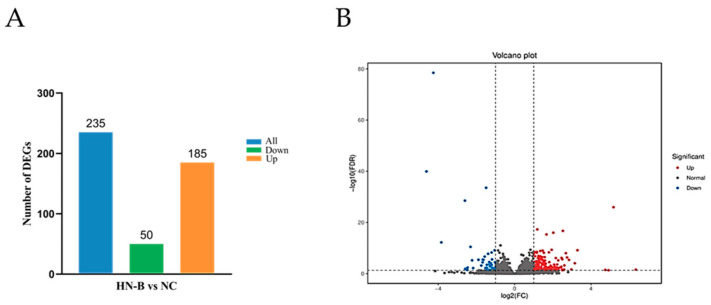
DEGs in HN-B vs. NC. (**A**) Bar graph of DEGs in HN-B vs. NC. Blue, green, and orange indicate the total detected DEGs, down-regulated DEGs, and up-regulated DEGs, respectively. (**B**) Volcano plot of DEGs in HN-B vs. NC. Red indicates up-regulated DEGs, blue indicates down-regulated DEGs, and gray indicates non-significant DEGs.

Among the raw data from the Illumina platform, sequences with adaptors and those of low quality were filtered out. The obtained valid data of each sample are listed in Table 2. The GC base ratios ranged from 49.86% to 50.35%. The Q20 and Q30 base ratios were all over 93.00%. In addition, the proportions of N bases were close to 0.00%. These results showed that the sequencing data were qualified, which ensured the quality of subsequent bioinformatic analyses. To acquire the DEGs between groups, the screening criteria were set as |FC| ≥ 2 and FDR < 0.05. We identified 235 DEGs in HN-B vs. NC (Figure 2). Notably, the DEGs were mainly up-regulated, indicating that the mice exhibited a positive transcriptional response to Mcc HN-B infection.

### 3.3. GO Enrichment Analysis

**Figure 3:**
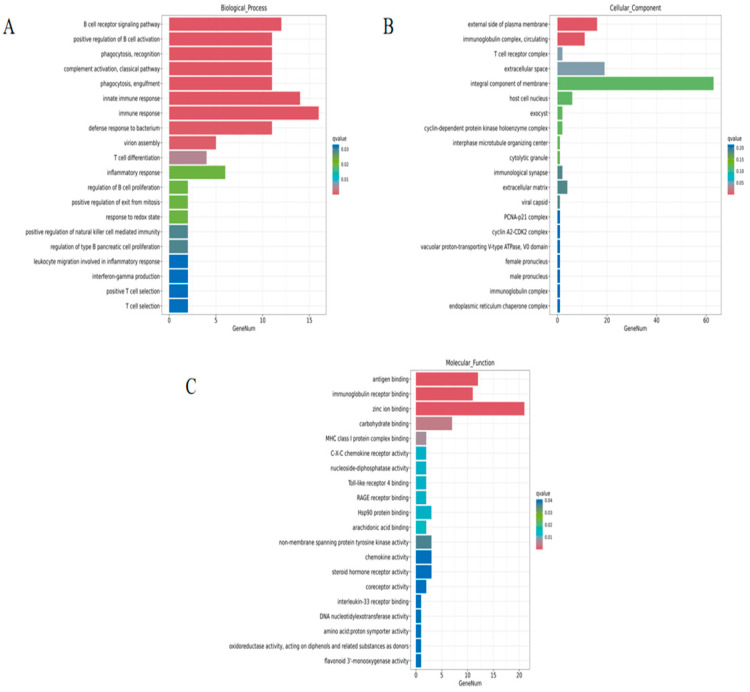
Bar plot of GO enrichment of DEGs in HN-B vs. NC. (**A**) Top 20 significantly enriched terms in biological processes. (**B**) Top 20 significantly enriched terms in cellular components. (**C**) Top 20 significantly enriched terms in molecular functions.

GO enrichment analysis showed that defensive response to bacteria, immune response, innate immune response, antigen binding, and immunoglobulin receptor binding were significantly enriched (Figure 3). In biological processes, immune response and innate immune response had a higher number of enriched DEGs, which were 16 and 14, respectively. Integral components of membrane contained the most DEGs in cellular components. As for molecular functions, the number of DEGs for zinc ion binding, antigen binding, and immunoglobulin receptor binding was relatively larger than for other terms.

### 3.4. KEGG Enrichment Analysis

**Figure 4:**
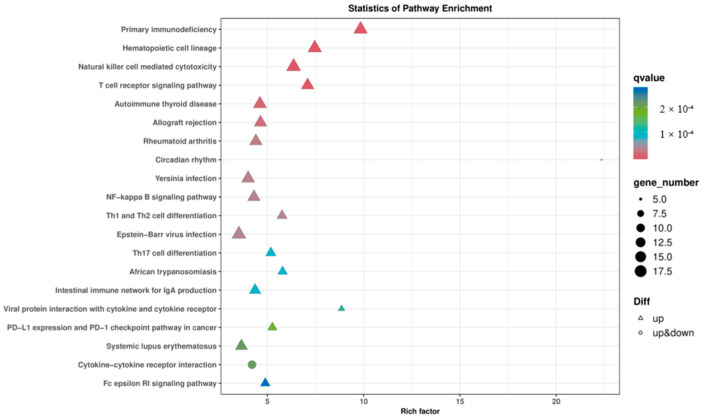
Scatter plot of the KEGG enrichment of DEGs in HN-B vs. NC. Top 20 significantly enriched pathways are shown.

According to KEGG enrichment analysis, the DEGs in HN-B vs. NC were primarily related to hematopoietic cell lineage, natural killer cell-mediated cytotoxicity, T cell receptor signaling pathway, NF-kappa B signaling pathway, Th17 cell differentiation, and cytokine–cytokine receptor interactions (Figure 4). In summary, the results manifested that multiple signaling pathways in the lungs of mice were affected during Mcc HN-B infection.

### 3.5. GSEA Enrichment Analysis

**Figure 5:**
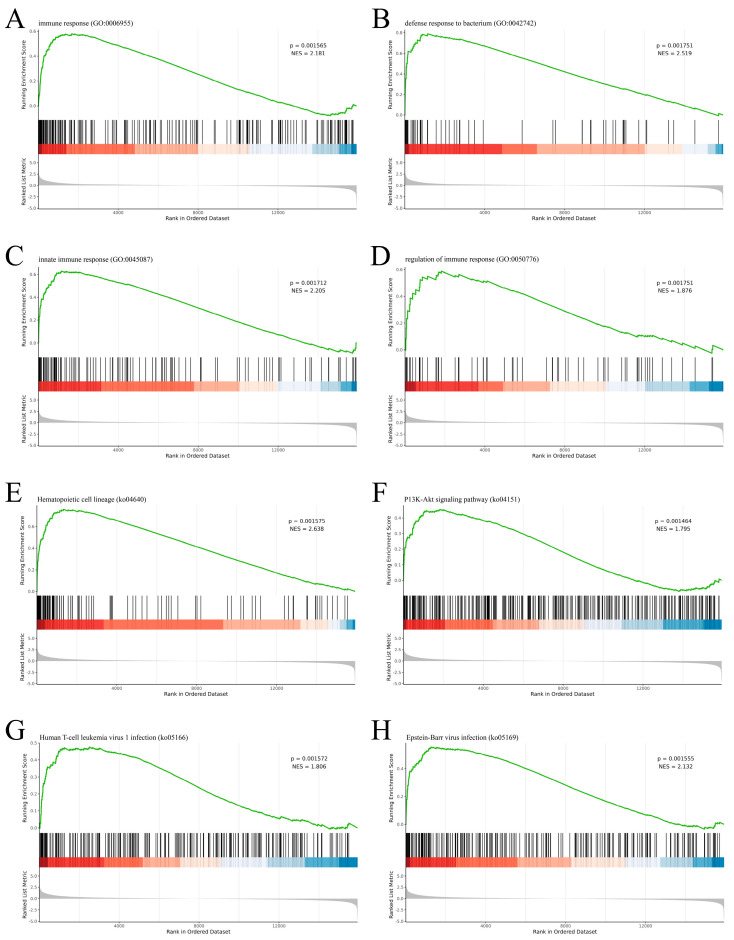
GSEA plot of GO terms and KEGG pathways. (**A**) Immune response; (**B**) Defense against bacteria; (**C**) Innate immune response; (**D**) Regulation of immune response; (**E**) Hematopoietic cell lineage; (**F**) PI3K-Akt signaling pathway; (**G**) Human T cell leukemia virus 1 infection; (**H**) Epstein–Barr virus infection. The green line represents the running enrichment score, which is calculated for each gene in the gene list and connected from left to right. The black vertical line indicates the position of each member of the gene list on the ordered dataset. Color bar from red to blue represents the relative expression (HN-B vs. NC) of genes in the gene list from increasing to decreasing. The bottom gray area plot shows the expression distribution of genes in the gene list.

GSEA analysis sheds light on the effect of overall DEGs on a specific phenotype. It showed that GO terms, including immune response, defense against bacteria, innate immune response, and regulation of immune response, were all up-regulated after Mcc HN-B infection (Figure 5A–D). Moreover, hematopoietic cell lineage, PI3K-Akt signaling pathway, human T cell leukemia virus 1 infection, and Epstein–Barr virus infection also exhibited an up-regulation trend (Figure 5E–H). These pathways potentially played a crucial role in resisting Mcc HN-B invasion. Noticeably, two virus-related pathways were also activated, which is consistent with the above findings.

### 3.6. PPI Network of DEGs

**Figure 6:**
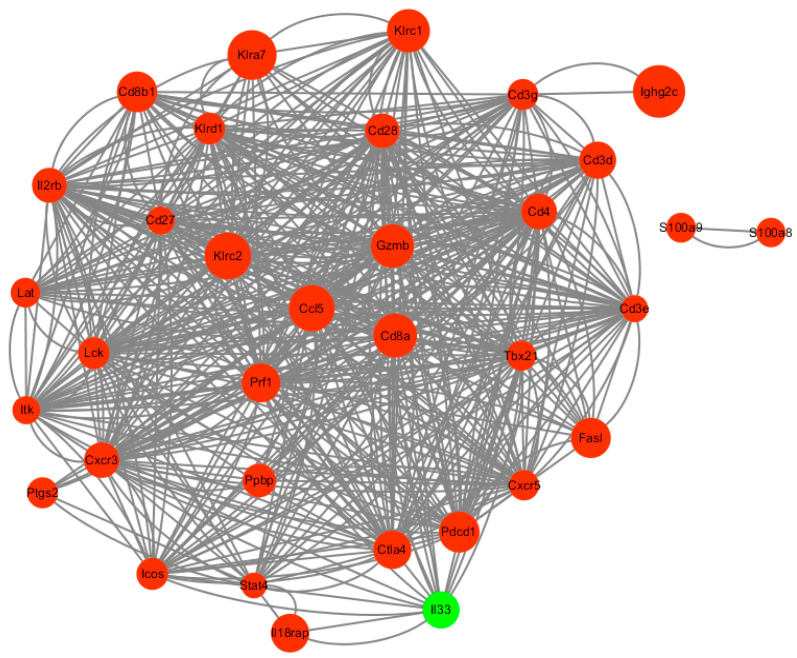
PPI network of the DEGs in immune-related pathways. Red and green represent up-regulated and down-regulated DEGs, respectively. Nodes indicate the proteins encoded by the corresponding DEGs. The size of the node represents the log_2_|FC| value of the DEG. The higher the value, the larger the node. Edges indicate protein–protein associations predicted by STRING.

The DEGs in immune-related signaling pathways, including IL-17 signaling pathway, Th1 and Th2 cell differentiation, NF-kappa B signaling pathway, natural killer cell-mediated cytotoxicity, T cell receptor signaling pathway, and cytokine–cytokine receptor interactions, were selected to perform a PPI network analysis. The results showed that the 34 proteins had complex interactions. All of the corresponding DEGs, except for *Il-33*, were up-regulated after Mcc HN-B infection (Figure 6). Notably, S100A8 and S100A9 were related to each other but did not interact with any other proteins in the PPI network.

### 3.7. RT-qPCR Validation of DEGs

**Figure 7:**
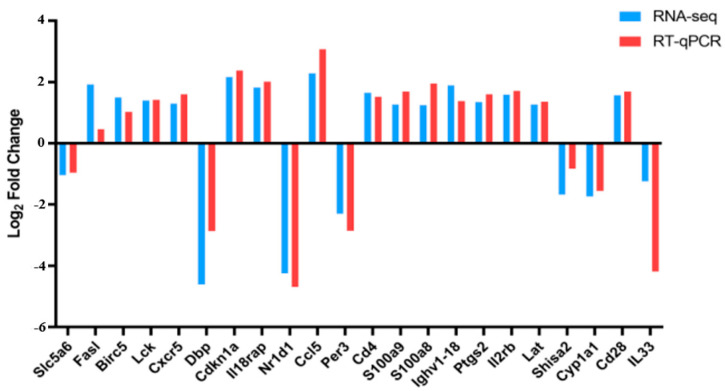
RT-qPCR validation of DEGs. Blue indicates the results of RNA-seq, while red indicates the results of RT-qPCR.

According to the quantitative results, 15 DEGs showed an up-regulated trend. The number of down-regulated DEGs was 7 (Figure 7). Moreover, these DEGs all exhibited a consistent expression trend in both RT-qPCR and RNA-seq.

### 3.8. Verification of the Expression of S100A8 and S100A9

**Figure 8:**
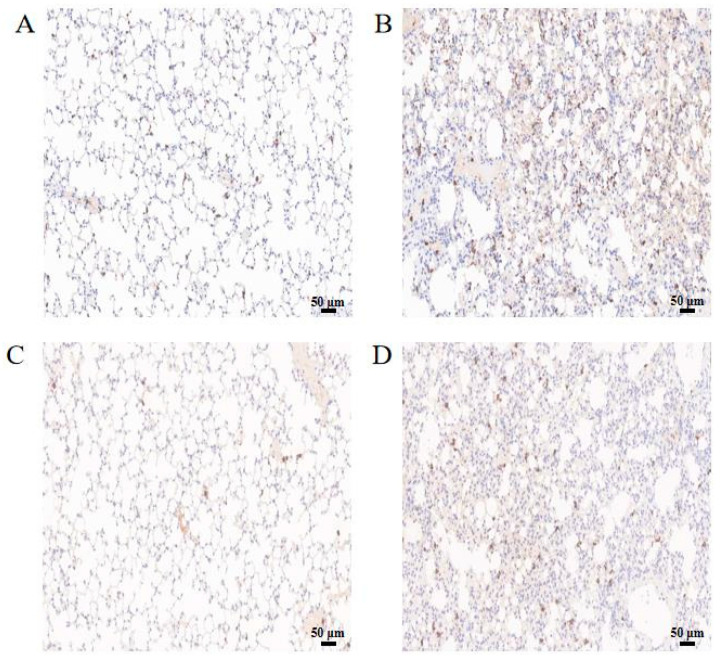
IHC of S100A8 and S100A9 in mouse lungs. (**A**) IHC of S100A8 in mouse lungs of the NC group; (**B**) IHC of S100A8 in mouse lungs of the HN-B group; (**C**) IHC of S100A9 in mouse lungs of the NC group; (**D**) IHC of S100A9 in mouse lungs of the HN-B group. Scale bar = 50 μm.

**Figure 9:**
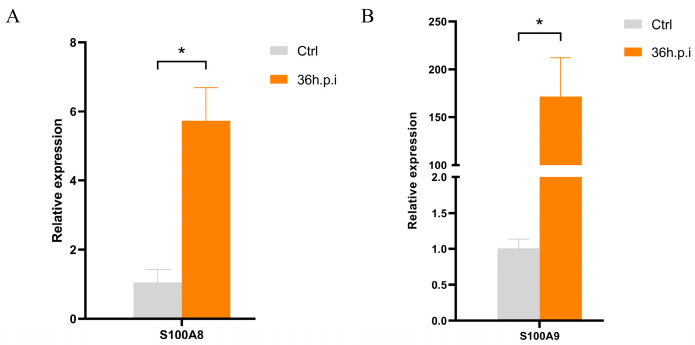
RT-qPCR analysis of *S100a8* (**A**) and *S100a9* (**B**) in Mcc HN-B-infected RAW264.7 cells. The data are represented as the mean ± SD. Two-tailed unpaired Student’s *t*-tests were used. * indicates *p* < 0.05.

The RT-qPCR results showed that *S100a8* and *S100a9* were up-regulated after Mcc HN-B infection (Figure 7). To verify their expression at the protein level, IHC was performed on the mouse lungs. As shown in Figure 8A,C, small amounts of S100A8 and S100A9 were present in the mouse lungs of the NC group. In the HN-B group, the expression of S100A8 and S100A9 was significantly elevated (Figure 8B,D). Further RT-qPCR assays on the Mcc HN-B-infected RAW264.7 cells also confirmed the up-regulation of *S100a8* and *S100a9* (Figure 9).

## 4. Discussion

In this study, we infected mice with Mcc HN-B to investigate its pathogenicity. The infection dose was 1 × 10^9^ CCU per mouse. During 3 days of continuous observation, the mice in the NC group behaved normally. However, the mice in the HN-B group showed depression and huddled together shortly after infection. At 6 h.p.i., these mice were in the lowest spirits and still huddled together. Meanwhile, loss of appetite and messy hair were observed. At 12 h.p.i., the mice in the HN-B group restored their appetite and began grooming, but they also exhibited clinical signs of tachypnea and lethargy. The mice were extremely excited at 24 h.p.i., which was manifested by rapid breathing, active feeding, and peculiar behavior of chasing and biting each other. No mice died in either of the groups within 3 days of observation.

Postmortem examination showed that the morphology of the heart, liver, spleen, and kidneys of mice in the HN-B group was normal compared to that of the NC group. Only partial lesions appeared in the lungs, accompanied by inflammatory exudate. Both autopsy and H&E staining suggested that Mcc HN-B caused lung inflammation and impaired alveolar ventilation, leading to shortness of breath. Moreover, the expression levels of two inflammatory proteins, S100A8 and S100A9, were elevated in the lungs of mice after Mcc HN-B infection. These results collectively indicated that Mcc HN-B induced pulmonary immune and inflammatory responses in mice.

By screening the RNA-seq data (|FC| ≥ 2, FDR < 0.05), a total of 235 DEGs were found in HN-B vs. NC. KEGG enrichment analysis suggested that Mcc HN-B activated multiple immune pathways in the lungs of mice. These pathways may play a crucial role during the process of resisting Mcc infection. RT-qPCR results showed that all 22 randomly selected DEGs had a consistent expression trend in both RT-qPCR and RNA-seq, confirming the reliability of the RNA-seq data. Significantly up-regulated DEGs included *Ccl5*, *Cdkn1a*, *Cxcr5*, *Cd4*, *Cd28*, *Il2rb*, *Lck*, *Lat*, *Ptgs2*, *S100a8*, and *S100a9*. In addition, *Il-33*, *Dbp*, *Nr1d1*, *Per3*, *Shisa2*, and *Cyp1a1* were found to be significantly down-regulated.

Interleukin-33 (IL-33) is considered to be a key factor in inducing Th2-type cytokine-associated immune responses, which plays an essential role in host defense against nematodes and allergic diseases [23]. When tissue is infected or damaged, it causes an inflammatory reaction to release an alarm signal [24]. Similarly to IL-1 and IL-18, IL-33 also serves as a pro-inflammatory cytokine to initiate non-Th2-type acute and chronic inflammation [23]. It has been found that IL-33 is responsible for mediating the Th2-type immune response in an allergic asthma model [25]. However, *Il-33* was significantly down-regulated in this study. We speculated that the expression of *Il-33* possibly varied at different stages of Mcc HN-B infection. During the first few hours of Mcc HN-B infection, IL-33 may be largely produced and cause pulmonary inflammation, which could explain the symptoms of depression and inappetence to some extent. In the late stage of infection, the mice had recovered mentally, and their symptoms were alleviated. Therefore, the expression of *Il-33* was inhibited to avoid severe inflammation and tissue damage.

C-C motif chemokine ligand 5 (CCL5) is a crucial element of the mammalian chemokine system. CCL5 is responsible for the migration and recruitment of T cells, dendritic cells, eosinophils, basophils, NK cells, and mast cells in vitro [26]. In the initial stage of viral replication, pro-inflammatory responses, which may lead to a prolonged course of disease, can be restrained by CCL5 [27]. More importantly, the activation of CCR5 by CCL5 plays a significant role in prolonging macrophage survival and controlling infection [28]. CCL5-activated CD4+ T lymphocytes, monocytes, NK cells, mast cells, and basophils are directly involved in the antiviral response [29]. In this study, the expression of *Ccl5* and *Cd4* was up-regulated. It may be reasonable that CCL5 was highly expressed in the lungs of mice and further activated CD4+ T lymphocytes, monocytes, and NK cells to fight against Mcc HN-B infection. This assumed process was similar to the above-mentioned antiviral effect of CCL5. Therefore, CCL5 is recommended to be a characteristic marker of Mcc infection in the host. The protein encoded by the inflammation-related gene *Il2rb* functions in the cell signal transduction pathway. It is closely related to the proliferation and differentiation of T lymphocytes and participates in the regulation of the immune system [30]. Interestingly, the expression of *Il2rb* was also up-regulated after Mcc HN-B infection. The specific role of *Il2rb* in Mcc infection needs further investigation.

In addition, LCK plays a key role in T cell receptor (TCR) signal transduction and CD28-dependent T cell activation [31,32,33]. It is constitutively associated with the CD4/CD8 co-receptors of thymocytes [34]. Meanwhile, LAT is a negative regulator of TCR signaling and T cell homeostasis [35]. The high expression of *Lck*, *Lat,* and *Cd28* is of great significance for regulating the differentiation, signal transduction, and homeostasis of T cells. In particular, these molecules have the potential to affect the specific elimination of pathogens and mitigate the inflammatory response in the late stage of Mcc HN-B infection.

Prostaglandin–endoperoxide synthase (PTGS), also known as cyclooxygenase, consists of two isoforms, namely, constitutive PTGS1 and inducible PTGS2 [36]. PTGS2 is induced by pro-inflammatory cytokines and participates in biological processes such as inflammatory response and apoptosis [37]. The heterodimer S100A8/A9, composed of S100A8 and S100A9, is involved in the arachidonic acid metabolism of neutrophils and monocytes [38]. Additionally, it can serve as a candidate diagnostic biomarker for inflammation-related diseases such as bacterial infection and autoimmune diseases [39]. The expression of S100A8/A9 in the infection-induced inflammatory response is limited by a negative feedback regulation mechanism. Only an appropriate amount of S100 family proteins is conducive to the anti-invasion ability and immune homeostasis of the host [19,40]. S100A8/A9 plays an important role in protecting the body from pathogen infection through multiple inflammatory pathways mediated by TLR4 or RAGE [41]. In this study, the high levels of *Ptgs2*, *S100a8,* and *S100a9* strongly indicated that an intense immune response occurred in the lungs of mice. The production of inducible PTGS2 and S100A8/A9 possibly contributed to the development of inflammation, which, in turn, led to serious tissue damage. However, due to the negative feedback regulation of S100A8/A9, more severe tissue damage and cytokine storms did not happen in mice, thus enabling them to survive after Mcc HN-B infection.

Further in vitro experiments demonstrated that *S100a8* and *S100a9* were significantly up-regulated in RAW264.7 cells after Mcc HN-B infection (36 h.p.i.), which was consistent with the results observed in the Mcc-infected mouse lungs. This not only shows that Mcc HN-B induced similar immune responses in both mice and RAW264.7 cells but also highlights the potential diagnostic value of S100A8 and S100A9 in Mcc infection.

## 5. Conclusions

In this study, RNA-seq, H&E staining, and immunohistochemistry were performed on Mcc HN-B-infected mice for the first time. The results showed that Mcc HN-B induced pulmonary inflammation and lesions. Up-regulation of S100A8 and S100A9 was verified in the lungs of the mice. Moreover, RT-qPCR showed that the expression of *S100a8* and *S100a9* was elevated in RAW264.7 cells after Mcc HN-B infection. S100A8 and S100A9 not only have diagnostic value in Mcc infection but also hold great significance in clarifying the pathogenic mechanism of Mcc.

## Figures and Tables

**Table 1:** Genes and primer sequences.

Genes	Forward Primer (5′–3′)	Reverse Primer (5′–3′)
*β-actin*	ACT GTC GAG TCG CGT CCA	ATC CAT GGC GAA CTG GTG G
*Slc5a6*	GCC CCT GAC CAG TTA GTC CT	AGA GAC AGG CAA CGA AGA GC
*Fasl*	CAC TAC CAC CGC CAT CAC AAC	TCC AAC CAG AGC CAC CAG AAC
*Birc5*	ATG ACA ACC CGA TAG AGG AGC	TTC TTG GCT CTC TGT CTG TCC
*Lck*	GCA CGA TCT AGT CCG CCA TTA CAC	CAG CCG CTC CAC CAA CTT CAG
*Cxcr5*	ATG AAC TAC CCA CTA ACC CTG G	GCC AGT TCC TTG TAC AGG TCA T
*Dbp*	GAT CTC GCC CTG TCA AGC ATT CC	TTC CTC CTC TGA GAA GCG GTG TC
*Cd28*	TTC TCA GTT CAA GTA ACA GAA AAC A	AGG CTG ACC TCG TTG CTA TC
*Shisa2*	ACA ACG ACC GCC AGC AG	ACG TAG ATG GGG ACT GCC GA
*Lat*	ATG GAA GCA GAC GCC TTG AG	GGG GGA CGG TTA TTT GAG GTG
*Cdkn1a*	GAT ATC CAG ACA TTC AGA GCC AC	ACG AAG TCA AAG TTC CAC CGT
*Il18rap*	GGA AAG CCT TTA ACT CTC CCC TG	AGG ATG TAT ACA AAC ACC ACC TCT
*Nr1d1*	GTG TAA GGT GTG TGG GGA CG	CCC AAA ACG CAC AGC ATC TCT A
*Ccl5*	TCC AAT CTT GCA GTC GTG TTT G	AGG GAA GCT ATA CAG GGT CAG
*Per3*	ACA ACT GGA CCA TCC ACA GAC	GGC AAC ACT TTC TGC TGA CTG
*Cd4*	AGC ATG TCA GGG GCA AAT GA	TGG CTT GGA TGT GTG TTG GT
*S100a9*	AAG AAA GAG AAG AGA AAT GAA GCC	TTG CCA TCA GCA TCA TAC ACT C
*S100a8*	ATG CCC TCT ACA AGA ATG ACT	TCA CCA TCG CAA GGA ACT
*Ighv1-18*	GAA CTG CAG GTG TCC TCT CTG	AGC CTT GCA GGG TAT CTT CAC
*Ptgs2*	TGC CCG ACA CCT TCA ACA TT	CAG CCA TTT CCT TCT CTC CTG T
*Il* *-* *33*	CAG CTA TTT CCT GTC TGT ATT GAG	TGG TCT TCT GTT GGG ATC TTC T
*Il2rb*	CAA CTC CAT GTT GCA GCC AG	GGT TTT GTT CCA GTG TCG CA
*Cyp1a1*	GTG GAG CCT CAT GTA CCT GGT AAC	TGC CGA TCT CTG CCA ATC ACT G

**Table 2:** Sequencing data statistics of each sample.

Sample Name	Clean Reads	Total Bases	GC Bases Ratio	Q20 Bases Ratio	Q30 Bases Ratio	N Bases Ratio
HN-B-1	20,645,123	6,176,754,998	50.02%	97.83%	93.77%	0.00%
HN-B-2	22,738,027	6,803,640,286	50.07%	97.89%	93.91%	0.00%
HN-B-3	20,780,502	6,218,888,444	49.89%	97.90%	93.96%	0.00%
NC-1	21,540,223	6,446,375,978	49.86%	97.75%	93.62%	0.00%
NC-2	23,047,209	6,895,583,090	50.35%	97.89%	93.95%	0.00%
NC-3	21,445,963	6,416,083,748	50.11%	97.97%	94.19%	0.00%

Note: Q20 and Q30 represent base calling error rates of 1% and 0.1%, respectively. N represents unknown bases. GC, Q20, Q30, and N base ratios all refer to their proportions in clean reads.

## Data Availability

The RNA-seq data in this study were deposited in the Genome Sequence Archive of China, National Center for Bioinformation (https://ngdc.cncb.ac.cn/gsa, accessed on 19 December 2023), under the accession number CRA014709.
